# A survey of urban climate change experiments in 100 cities

**DOI:** 10.1016/j.gloenvcha.2012.07.005

**Published:** 2013-02

**Authors:** Vanesa Castán Broto, Harriet Bulkeley

**Affiliations:** a Development and Planning Unit, UCL, 34 Tavistock Square, London, United Kingdom; b Geography Department, Durham University, Science Site, South Road, Durham, United Kingdom

**Keywords:** Climate change experiments, Mitigation, Adaptation, Governance, Cities, Infrastructure

## Abstract

Cities are key sites where climate change is being addressed. Previous research has largely overlooked the multiplicity of climate change responses emerging outside formal contexts of decision-making and led by actors other than municipal governments. Moreover, existing research has largely focused on case studies of climate change mitigation in developed economies. The objective of this paper is to uncover the heterogeneous mix of actors, settings, governance arrangements and technologies involved in the governance of climate change in cities in different parts of the world.

The paper focuses on urban climate change governance as a process of experimentation. Climate change experiments are presented here as interventions to try out new ideas and methods in the context of future uncertainties. They serve to understand how interventions work in practice, in new contexts where they are thought of as innovative. To study experimentation, the paper presents evidence from the analysis of a database of 627 urban climate change experiments in a sample of 100 global cities.

The analysis suggests that, since 2005, experimentation is a feature of urban responses to climate change across different world regions and multiple sectors. Although experimentation does not appear to be related to particular kinds of urban economic and social conditions, some of its core features are visible. For example, experimentation tends to focus on energy. Also, both social and technical forms of experimentation are visible, but technical experimentation is more common in urban infrastructure systems. While municipal governments have a critical role in climate change experimentation, they often act alongside other actors and in a variety of forms of partnership. These findings point at experimentation as a key tool to open up new political spaces for governing climate change in the city.

## Highlights

► A database analysis reveals urban climate change experimentation as a global trend. ► Experimentation is a recent trend not confined to specific world regions or cities. ► Although experimentation is heterogeneous, energy experiments predominate. ► Multiple actors, often through partnership, intervene in urban climate change governance. ► A characteristic trend of experimentation led by private actors emerges in Asia.

## 1 Introduction

Addressing climate change requires an “unprecedented level of cooperation, not only between countries, but also between different levels of Governments and the private sector” (De Boer, 2009, p. 1). The city is an increasingly important site for climate response. While there remains much dispute about the exact contribution that cities make to GHG emissions (Dodman, 2009), and about who and what is most vulnerable to the effects of climate change (De Sherbinin et al., 2007), urban centres are now regarded as a vital part of the global response to climate change (UN-Habitat, 2011; World-Bank, 2010).

While recognition of urban responses to climate change at the international policy level has been relatively recent, a burgeoning research community has studied the relationships between cities and climate change. Since the mid-1990s, research has focused on municipal strategies, policies and measures, and the challenges that municipal authorities face in terms of policy implementation and effectiveness. This body of work, mainly developed with case-study methods, has yielded numerous insights including: the multiple modes of governing through which municipalities seek to govern climate change; the importance of institutional capacity, including resources, knowledge and organisational structures; the critical role of individuals, political champions and policy entrepreneurs; and how multi-level governance structures opportunities and limits for municipal action (see Betsill and Bulkeley, 2007; Bulkeley, 2010; Schreurs, 2008 for recent reviews). However, this work also has limitations to understand how, why and with what implications urban responses to climate change are taking place.

The first issue concerns the *type* of studies and cities studied. Research has mainly focused on generating rich data about either individual case studies or small sets of cities. Such approaches, combined with a focus on early city pioneers and members of specific transnational municipal networks, have created a geographical bias towards cities in more economically developed countries, predominantly the US, Canada, Europe and Australia (e.g. Allman et al., 2004; Bulkeley and Betsill, 2003; Bulkeley and Kern, 2006; Davies, 2005; Kousky and Schneider, 2003; Lindseth, 2004), although there are now an increasing number of cases in Asia and Latin America (Bai, 2007; Dhakal, 2006; Holgate, 2007; Ranger et al., 2011; Romero Lankao, 2007). Moreover, research has primarily focused on mitigation, rather than adaptation (see recent exceptions Hallegatte and Corfee-Morlot, 2011; Hunt and Watkiss, 2011; Romero-Lankao and Dodman, 2011; Romero Lankao, 2007; Satterthwaite et al., 2009). Fewer studies have sought to undertake systematic comparison between cases, or have employed quantitative methodologies. Where these exist, analysis has focused on whether particular urban characteristics explain the emergence of particular kinds of policy response within cities in more developed economies (e.g. Krause, 2011; Pitt and Randolph, 2009; Zahran et al., 2008). Overall, our understanding of urban responses to climate change is largely derived from case-study work, focused on cities in more developed economies and mitigation responses.

A second limitation has been the predominant concern with understanding the role of local authorities in shaping urban responses. The literature on global environmental governance now makes clear that non-state actors (corporations, NGOs, international foundations, community groups) are increasingly involved in responding to climate change (Bulkeley and Newell, 2010). Moreover, the boundaries between the public and private actors are increasingly blurred, as private organisations take on roles traditionally regarded as the province of the state, while public authorities are engaged in forms of activity often regarded as a private domain, such as intervening in carbon markets or promoting the energy economy. These coupled issues – the growing roles of private actors in responding to climate change and the blurring of the public/private boundary – mean that it is no longer sufficient to regard urban responses to climate change as a matter for municipalities alone.

A third limitation to our current understanding of urban responses to climate change is the analytical focus on the processes of agenda setting and policy-making, the development of plans and strategies and the selection of specific measures in different contexts. Less attention has been paid to responses to climate change taking place outside of formalised policy channels, constraining our knowledge of these interventions.

A fuller understanding of urban responses to climate change will require new forms of case-study and comparative research that consider a more geographically diverse range of cities together with the range of urban actors involved in such responses, and capture initiatives and interventions falling outside of formal processes of planning and policy. In this paper, we discuss the results from one methodological approach – a survey of the climate change initiatives or experiments taking place in 100 cities – designed to further this agenda. Despite the acknowledgement that there remains a ‘stubborn gap’ between the rhetoric and reality of local climate policy and its implementation (Betsill and Bulkeley, 2007), urban landscapes are littered with examples of actions being taken under the banner of climate change. Our approach examines these initiatives, which we term ‘climate change experiments’.

The concept ‘climate change experiment’ (Bulkeley and Castán Broto, 2012) is based on insights from literatures on governance experiments (Hoffman, 2011), the role of niches and grassroots innovations in socio-technical regimes (Geels et al., 2011), and the notion of ‘urban laboratories’ (Evans, 2011) that point to the ways in which experimentation forms part of the governance and contestation of socio-technical systems. We define urban climate change experiments according to three criteria which build upon these perspectives: first, an intervention is experimental when it is purposive and strategic but explicitly seeks to capture new forms of learning or experience; second, an intervention is a climate change experiment where the purpose is to reduce emissions of greenhouse gases (mitigation) and/or vulnerabilities to climate change impacts (adaptation); third, a climate change experiment is urban when it is delivered by or in the name of an existing or imagined urban community. Climate change experiments are presented here as interventions to try out new ideas and methods in the context of future uncertainties. They serve to understand how interventions work in practice, in new contexts where they are thought of as innovative.

The objective of the research was to understand the extent and diversity of climate change experimentation both in the global north and the global south adopting a comparative approach to capture the extent and diversity of urban climate change experiments. The analysis considered: when and where urban climate change experiments emerge; what types of urban climate change experiments we find and what are their characteristics; and who leads these experiments and what mechanisms make them possible. Results suggest that experimentation is a feature of urban responses to climate change across different world regions and multiple sectors but it does not appear to be related to particular kinds of urban economic and social condition. Some core features of experimentation are visible. Experimentation, like other forms of urban climate change response, tends to focus on energy. Both social and technical forms of experimentation are emerging, though the latter is most common and dominates the urban infrastructure systems within which experimentation is most common. Municipalities have a critical role in experimentation, though analysis also reveals the wide variety of forms of partnership through which experimentation is taking place and that are arguably opening up new political spaces for governing climate change in the city.

## 2 Methodology

The construction of the database involved surveying 100 cities using secondary materials, and the systematic storage of information to facilitate the analysis. The construction of the database involved a selection of cities, database design, data collection and analysis.

### 2.1 Selection of cities

In academic discourse, ‘global city’ refers to cities that are important nodes within the global economic system (Sassen, 1991), but colloquially it also refers to cities that have significance because of their size and concentration of population, or political significance. The sample in this research was designed to represent a sample of a heterogeneous group of cities in all parts of the world with clear significance in terms of contributions to greenhouse gases and concentration of vulnerabilities to climate change, using six indicators: Total Population and Density indicate the extent to which exposure to climate vulnerabilities may be concentrated in the urban arena and the potential total GHG emissions from any one city or urban area. Indicators of economic activity were used as a proxy to reflect the overall contribution to GHG emissions, including gross domestic product and a ‘world city’ indicator to characterise cities that have an established role in international economic networks providing global service centres and graded for accountancy, advertising, banking/finance and law (Beaverstock et al., 1999). Two other indicators were introduced, one to select all cities which actively participate in the C40 Climate Leadership Group, and another to highlight cities with specific vulnerabilities to climate change, including, port cities, cities vulnerable to sea level rise (Nicholls et al., 2008; UN-HABITAT, 2008) and cities vulnerable to glacier changes (Stern et al., 2006). Data was obtained from the City Mayors website (City Mayors, 2012). Six hundred and fifty cities were ranked according to the indicators, and all ranks were added to establish a compound measure for each city. The final sample included the top one hundred cities, which scored relatively high in all indicators, but with clear variation among the cities for all indicators (Tables 1 and 2).

### 2.2 Database design

Each record in the database corresponds to a discrete urban climate change experiment. Following previous comparative research about municipal responses to climate change in eight cities (Bulkeley et al., 2009; World-Bank, 2010) the database was divided in six sheets, one for each of five key sectors of climate change mitigation (urban infrastructure, built environment, transport, carbon sequestration and urban form) and one for adaptation experiments (see Table 3).

Analytical categories recorded in each record cover: (1) where and when urban climate change experiments occur; (2) what are these experiments how are they developed and (3) who leads initiatives and how they are governed (Table 4).

Indicators of where and when urban climate change experimentation occurs provide a sense of the context in which these initiatives occur. Each initiative was dated in relation to the approval of the Kyoto protocol in 1997 and its ratification in 2005.

Recording specific types of innovation was a means to check that the initiative met the definition of experiment and provided a ground for comparison, as experiments reflected attempts to develop technological innovations (designs, technologies, materials), social innovations (policy tools, financial mechanisms, changes to cultural norms) or both. The form of innovation was a better indicator than the factors which made the experiment possible, because while the form of innovation was always reported, the factors leading to the experiment were not always explicit or were only found in secondary sources. For each sector the database included specific aspects of the system of provision in which the experiment intervened (see Table 3) and the specific service which was met.

The design follows an understanding of governance as a multi-level and multi-actor process. The database captured the experiment leading actors, but also recorded separately the partnerships that made the experiment possible. The information regarding funding mechanisms and costs was very fragmentary. Modes of governance were also recorded. A mode of governance is a set of tools and technologies deployed through particular institutional relations through which agents seek to reconfigure the specific social and technical relations with a specific governing purpose (Bulkeley and Kern, 2006), in this case, to address climate change. Municipalities can deploy four modes of governance including: (1) self-governing, intervening in the management of local authority operations to “lead by example”; (2) provision, greening infrastructure and consumer services provided by different authorities; (3) regulations, enforcing new laws, planning regulations, building codes, etc.; and (4) enabling, supporting initiatives led by other actors through information and resource provision and partnerships (Bulkeley and Kern, 2006). Given that climate change action requires coordination of mutually dependent actions beyond public institutions (Bulkeley et al., 2009; Kern and Alber, 2008), this concept was extended to non-governmental actors leading climate change experiments.

### 2.3 Data collection methods

Information on experiments was collected through three main means: review of key literature; consultation with climate change experts; and Internet searches. Interviews with individuals at the International Institute of Environmental Development, the Building and Social Housing Foundation (including access to their large database of innovation projects in housing worldwide) and urban experts at the World Bank provided examples of experimental initiatives which are considered to be leading worldwide. Internet searches looked systematically through the websites of local, regional and national governments and private and civil society organisations, news items and reports for each city in turn. Additional data was obtained from the Clean Development Mechanism database (UNFCCC, 2012). The search looked beyond recognised examples of best-practice and recorded as many instances of experimentation as possible in an allotted amount of time. The archival system included a folder per city with a city-specific summary of the main climate change activities, a list of experiments recorded in the database and a collection of data sources backing the information provided in the database records.

The data was compiled from June 2009 to June 2010, with a revision and update of data in December 2010. The predominant use of Internet data sources had some limitations because it relied in self-reported data. Self-reported data may focus on making the experiment rather than its implementation in practice and it is more likely to report successes than difficulties and failures. Moreover, many interesting experiments may not be reported on the Internet or may be inaccessible to standard search engines. Overall, there were practical limitations in terms of the time dedicated to each city (we dedicated in average 2 days per city but included additional time for cities where less information was available) and the languages covered (the database included initiatives reported in Portuguese, Spanish, English, French, Italian and German but crucially, not those in key languages such as Chinese and Russian). Thus, the database should not be regarded as comprehensive, but rather, as providing an indicative account of the emergence of climate change experimentation in these cities.

### 2.4 Analysis of database results

To facilitate the statistical analysis, we re-coded numeric dates in reference to the approval and ratification of the Kyoto protocol; the type of innovation to register whether the experiment included technological innovation, social innovation or both; the schemes used, focusing on the interventions on energy systems and whether the experiment was directed at producers (energy generation and transmission measures) or at consumers (demand side measures); and the type of actors as public, including local government, regional government, national government, international organisation, private and civil society organisations, including non-governmental organisations (or charities) and community-based organisations. Variables for which information was incomplete or unconfirmed were excluded.

We also used the city-based variables (see Table 2) and a variable registering cities’ membership to the following transnational municipal networks:

• ICLEI, Local Governments for Sustainability, an association of over 1200 local governments working for sustainability which work together since 1990.
• Cities for Climate Protection, an affiliate programme of ICLEI in which cities commit to concrete actions for carbon reduction.
• C40 Cities Climate Leadership Group (C40), a network of cities created in 2005 by the London Mayor and the Clinton Foundation's climate change initiative.

The analysis examined: (1) where and when urban climate change experiments occur; (2) what are these experiments how are they developed and (3) who leads initiatives and how they are governed. Variable comparison used either linear regression or correlation statistics in the case of categorical variables.

This approach advances and complements existing studies because it develops a large-n sample, in contrast to case-study work; it works with a variety of urban contexts, north and south, unlike previous survey-based analyses focused on one national context; and it focuses on climate change experiments, rather than plans and policies. The limitations of the study are in terms of sacrificing breadth for depth, both in understanding each experiment and exploring richer data that emerge from research in specific locations.

## 3 Results and discussion

The results concern three main questions: (1) where and when these experiments occur; (2) what types of interventions are emerging as climate change experiments and the extent to which we can identify some common trends and characteristics; and (3) who leads the experiments and what governance mechanisms make them possible.

### 3.1 Where and when do these experiments emerge?

Most experiments in the database, that is 79% of them (495 experiments) started after 2005, that is, after Kyoto was ratified. Only 5% of initiatives started before its initial adoption in 1997. This is not necessarily an indication that international agreements have direct impact in fostering climate change experimentation, but rather, that international climate change governance efforts correspond with an increasing interest on climate change in the collective imaginations of urban actors. Climate change has gained more visibility in the city at the same time as the agreements took place (Hoffman, 2011).

**Fig. 1:**
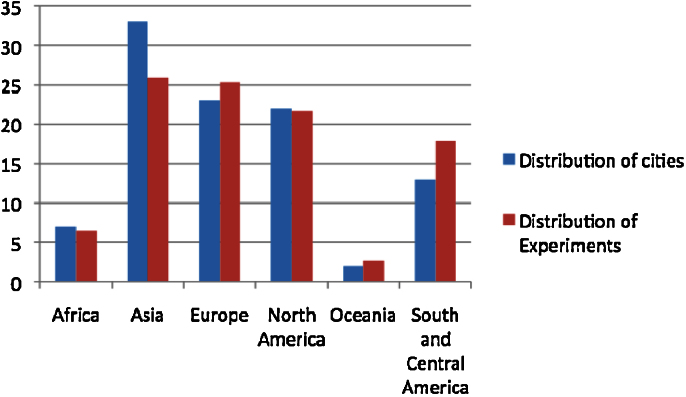
Comparison of the frequency distribution of cities and experiments in different world regions.

The observed frequency of experiments in all world regions is a function of the distribution of cities in the sample (Fig. 1), an observation confirmed by the statistical correlation test. This suggests that urban climate change experiments are not necessarily confined to certain world regions, such as, Europe and North America.

We also examined the association between urban climate change experiments in “more developed”, “less developed” and “least developed” nations (UN, 2010). The distribution of experiments is similar to the distribution of cities in world regions, with 8 experiments in cities in least developed regions (2%), 291 (46%) in less developed ones and 328 (52%) in more developed regions. The statistical correlation test confirms that the distribution of the sample of experiments is a function of the selection of cities, supporting the conclusion that urban climate change experimentation is not confined to any regions of the world.

The analysis also looked into what urban characteristics predict the emergence of experiments. The total number of experiments found in each city was taken as the dependent variable, and independent variables included those whose data was compiled during the selection of cities (Total Population, Total GDP, World City Rank and Density and adding Total Land Area, GDP per capita and Annual Population Growth). We applied a linear regression model using different combinations of variables, from one up to seven. The best goodness of fit model was a model that included the seven variables, but the statistics for the model suggest that the predictive value of the model is limited. Whether a city is richer, or more populated or denser does not predict accurately whether we are more likely to find more experiments in such a city.

An alternative hypothesis is that experiments as more likely in cities involved in transnational municipal networks, an important institutional arrangement through which climate change is governed (Kern and Bulkeley, 2009). Belonging to a network often requires taking certain forms of action, from plans to direct commitments, to reduce emissions or improve adaptation. The test evaluated to what extent the number of experiments in a city (dependent variable) could be explained by whether or not a city belonged to any of these networks. An independent variable was defined by whether or not a city belonged to transnational municipal networks. When we considered this variable together with the seven variables described above it improved the goodness of fit of the overall model, suggesting that this influences whether urban climate change experimentation is likely to occur and/or be more visible (although this comment should be taken with caution, considering that the model only explains 63% of observed values). The analysis of correlation between variables shows that the variable of whether or not the city belongs to a city network has a stronger association with the number of experiments in each city than any of the other variables described above. The importance of transnational municipal networks confirms the findings of case studies of urban climate governance. For example, London's prominent role as a site of experimentation (Hodson and Marvin, 2007; Bulkeley et al., 2012) has been supported by its active role in the C40 network. Yet, urban climate change experimentation goes beyond international policy initiatives, size and concentration of resources or population. Understanding the drivers and nature of urban climate change experimentation requires a more fine grained analysis, including looking into the kind of experimentation that occurs and how it is governed, the two issues that are analysed in turn in the following two sections.

### 3.2 What types of climate change experiments can we find and what are their characteristics?

**Fig. 2:**
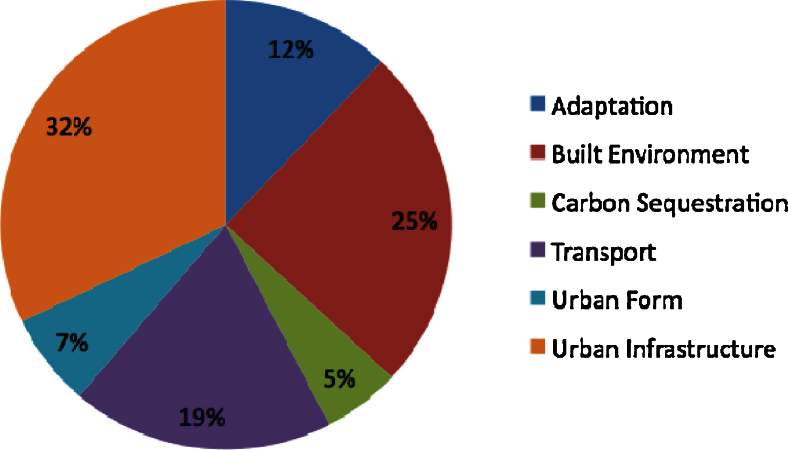
Distribution of experiments in sectors.

Most experiments are in the sectors urban infrastructure (31.1%), built environment (24.7%), and transport (18.8%). Adaptation experiments only account for 12.1% of the initiatives (Fig. 2).

Adaptation initiatives may be less represented in the database because they have less visibility as experiments than those concerned with mitigation. Adaptation initiatives focus on taking anticipatory action to deal with future climate risks. Different areas of intervention for climate change adaption include protection (e.g. vulnerability assessment, capacity building and risk reduction measures); pre-disaster damage limitation (e.g. early-warning systems and community-based disaster preparedness and response plans); immediate post-disaster responses (rapid infrastructure restoration); and rebuilding (Moser and Satterthwaite, 2008). However, adaptation is often regarded as a transversal issue to be considered in most operations and not always differentiated from on-going development efforts or disaster management programmes (Satterthwaite et al., 2009). Because many adaptation initiatives are not necessarily taken purposively in the name of climate change, they are therefore missing from our definition of climate change experiments.

Urban climate change experiments concentrate in urban infrastructure despite the difficulties inherent to manage infrastructures at the local level. Built environment and transport experiments are frequent in cities in the South were rapid population growth in peri-urban areas has led to raising demands for housing and transport (Allen, 2003). Less frequent are urban form and carbon sequestration experiments. In the case of urban form, one possible explanation is that there are still few practical examples of how to address mitigation through planning (but see Davoudi et al., 2009). The absence of carbon sequestration experiments highlights that either cities lack land resources to implement large carbon sequestration programmes or urban greening programmes are developed with independence of concerns with climate change mitigation.

**Fig. 3:**
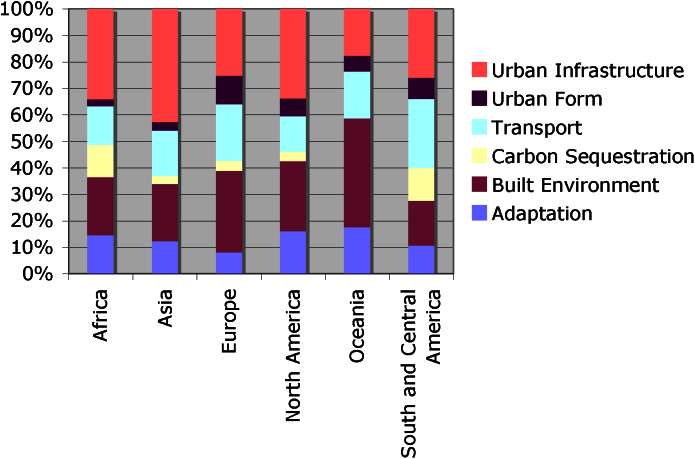
Frequency of experiments in different sectors in different regions of the world.

Fig. 3 provides an overview of the relative frequency of experiments in each sector in the different world regions considered above. The graph shows that although experiments in all sectors were found in every region, certain sectors appear to predominate in some areas. For example, in Asia, the data suggest that urban infrastructure experiments are more frequent. Transport projects are more popular in Central and South America, reflecting the regional impact of flagship transport experiences in Curitiba (Brazil) and Bogotá (Colombia) (Arup, 2011).

Table 5 presents demonstrates the association between sectors, time periods and regions. As experiments concentrate in the last period since the ratification of the Kyoto protocol, the subsequent hypothesis is whether this is reflected in the growth of experimentation across sectors. The statistical test of independence suggests that there is no association between the sector and the time of occurrence.

The second half of the table shows the total number of experiments in each sector in either less or more developed regions, to explore the association between the sector of urban climate change experimentation and different levels of development. Because of the distribution of the data, least developed and less developed regions were grouped together (least developed regions are defined as a subset of less developed ones, see UN, 2010). The test shows a weak association between the sectors and the regional distribution of experiments. Tests of association between specific regions and specific sectors suggest that while in most regions experiments are likely to emerge in any sector, in Asia, particularly, there is a predominance of urban infrastructure experiments. While it may be tentatively argued that the rapid processes of urbanization taking place in this region provide some degree of explanation for these findings, further research is needed to understand the broader drive in Asia towards this sector, and in particular, examining the flows of capital invested in large scale low carbon infrastructure.

Urban climate change experiments are socio-technical because they purposively attempt to change the material arrangements and the cultures, norms and conventions that determine collective GHGs emissions and climate-related vulnerabilities in the city (Bulkeley et al., 2011). For example, a survey of climate change plans in 30 cities worldwide identified the most common mitigation measures in transport (Wagner, 2009) including examples of experiments such as the congestion charge in London or the experimentation with new ideas about the provision of transport in the city or the use of alternative fuels in other European cities (see e.g. Bertaud et al., 2009; Leape, 2006; Prud’homme and Bocarejo, 2005).

Experiments challenge the technical basis of GHGs emissions, the social practices that produce them or both. Technical forms of innovation were more prevalent in the database, in 76% of all experiments (Table 6). Technical innovation was frequent in all sectors, especially in urban infrastructure, where 88% of interventions had a technical innovation component, but less frequent in carbon sequestration (40% of initiatives) and adaptation (60%). Social innovation was present in half of all the initiatives (50%). It was most frequent in carbon sequestration (60%) and urban form (64%) and most rare in urban infrastructure (39%). Is the type of innovation independent of the sector of intervention?

The test of independence between variables suggests that although social and technical innovations emerge in all sectors, technical innovation is more likely in urban infrastructure experiments, while social innovation is more likely in adaptation, carbon sequestration and urban form experiments. Built environment experiments favour interventions that combine both social and technical innovation. In transport experiments neither type is more prevalent.

Because of the strong links between energy use and GHGs emissions, urban climate change action has mostly focused in measures to optimise energy production, distribution and consumption. A study for the World Bank of climate change action in eight cities found that energy efficiency issues dominate the local agenda in climate change mitigation (Bulkeley et al., 2009). Improving the efficiency of appliances and designs is often coupled with behavioural measures to reduce energy demand (Betsill and Bulkeley, 2007).

The extent to which initiatives in these sectors focus on reconfiguring energy systems is reflected in Table 7. The majority of interventions in the built environment and urban infrastructure systems were explicitly concerned with intervening in the energy system (74.8% of initiatives in the built environment and 77.6% of initiatives in urban infrastructure). Energy related initiatives were less frequent in urban form interventions (only 9 initiatives). This confirms a common observation among local policy-makers (for example those involved in the well-known Climate Change Action Plan in Mexico City), about the lack of means to put into practice low carbon planning principles to address issues of density and urban form and the resulting emphasis on punctual projects in infrastructure and the built environment (Castán Broto, 2011).

Analyses of energy systems often tend to focus in the consumption or demand side, looking at energy end uses, and a production or supply side, looking at the generation and distribution of energy (RaEng, 2010). Table 7, an analysis of a sub-set of 281 experiments whose major objective is to intervene in energy systems, shows that most experiments in the database seek to intervene in energy consumption processes, although there is a trend towards new systems of energy production and generation in urban infrastructure, confirmed by the independence test. Since perceived size of investment and restructuring needed to develop a systemic change is a barrier to production-oriented interventions (RaEng, 2010), the emphasis on demand-side interventions may reflect greater possibilities to intervene in a distributed manner.

Overall, experiments constitute strategies to open up new forms of intervention in different urban spaces. Who has capacity and authority to intervene leading and participating in urban climate change experiments is the broader question of governance to which the following section turns.

### 3.3 Who leads these experiments and what mechanisms made them possible?

The analysis explored three aspects of urban climate change governance: the actors who lead action; the increased relevance of partnerships as a form of governance; the deployment of specific governance mechanisms, or modes of governance; and the extent to which environmental justice was a facet of experiments.

**Fig. 4:**
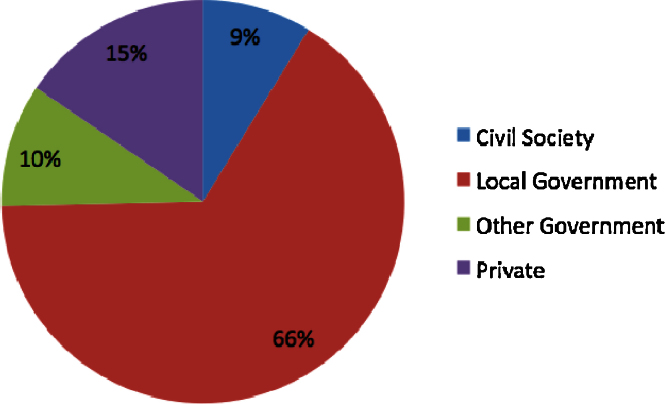
Distribution of frequency of different types of actors leading urban climate change experiments.

Fig. 4 shows that, in line with previously gathered evidence through case-study research, local governments have a prominent role in leading 66% of urban climate change experiments. However, the data also reveal that, alongside city governments, other actors may be playing a key role in climate change experimentation such as private and civil society actors.

Table 8 shows that actors are not confined to certain regions and there is variation in how actors operate. Using independence tests for each pair of values we established that, while in most cases the presence of an actor leading the experiment is independent from the region of operation, the tests of independence support the observation than private actors predominate in Asia, while other actors, especially civil society actors, lead fewer experiments than expected in that region. The predominance of private actors in Asia may be related to the rapid growth that has made capital available for climate change experiments, especially in infrastructure (see above). Private actors emerge as more likely to operate in capital-intensive sectors such as urban infrastructure while other actors do not have strong associations with any specific sector.

**Fig. 5:**
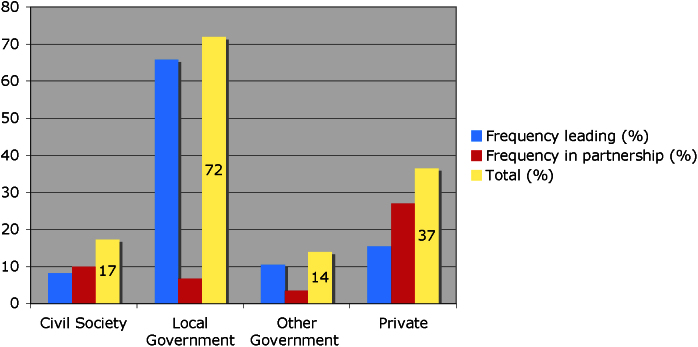
Frequency of actors in climate change experiments, either leading or as partners.

Partnerships are important for local governments because they extend the operation of the state through facilitating further action by other actors (Kern and Bulkeley, 2009). Beyond the local government, partnerships are generally considered a key tool for capacity building (Eakin and Lemos, 2006) and building consensus (Newman et al., 2009). In the database, 296 experiments (47%) involved some form of formally recognised partnership between actors at different governance levels, whether this is in terms of vertical governance (e.g. partnerships between local, regional and national governments) or horizontal (e.g. partnerships between governments, civil society organisations and private actors). When considering participation, rather than leadership, multiple actors gain prominence (Fig. 5).

Table 9 shows that the most common forms of partnership are those in which the local government leads with either private actors (112 experiments) or civil society actors (44 experiments). Local governments operate outside partnership more often than expected (in 239 experiments) whereas for other actors the frequency of operating in partnership is higher than expected. Civil society organisations often lead initiatives enrolling local governments as partners. This highlights that government support may be important in achieving projects led by civil society organisations, both in terms of providing resources and institutional support. Another significant trend is that private actors are able to draw partnerships with other private actors, for example, in partnerships between service delivery and financial organisations to make low carbon infrastructure projects possible.

Analysis of modes of governance throws further light in terms of how the governance of climate change is being performed. This theory was originally developed with reference to municipal organisations (Bulkeley and Kern, 2006; Bulkeley et al., 2009). So far, our results suggest that the realm of authority is being blurred both because of the prominence of partnerships and the increasing importance of non-governmental actors in areas traditionally considered as governed by governmental actors (Table 10). Tests of independence show strong association of the modes of governance with the leading actors and the emergence of partnerships. Partnership makes enabling initiatives more likely and regulation initiatives less likely (Table 10). Thus, enabling may be a tool for different actors to built explicit forms of support from other actors as a means for establishing authority beyond their own realm.

As the social and economic costs of climate change increase, attention is turned towards the equity implications of collective responses to climate change (Giddens, 2009). Climate justice debates are often framed in terms of nation-wide inequalities, and the responsibilities of industrialised countries in producing climate change. However, when examining the fabric of the city, it appears that the distribution of climate change responsibilities and vulnerabilities is often parallel to existing patterns of urban inequality (Satterthwaite, 2008). This raises questions about to what extent urban climate change experiments are concerned with justice and equity implications.

Environmental justice concerns were found in 154 climate change experiments (24.6%) and they were more common in urban form, built environment and adaptation.

A second concern is whether certain actors play a key role in advancing justice-related arguments. The contingency table (Table 11) shows that while both private actors and civil society organisations considered justice explicitly in their experiments, public actors were less likely to do so, which is confirmed by the strong association between the two variables. One explanation for the absence of justice claims in publicly led experiments is that government actors already operate under the belief of having the mandate to govern, which includes considerations of legitimacy and social justice, whereas private and civil society actors may make explicit environmental justice claims to justify their operations. Broader explanations pointing at the dominance of elites or the utilitarian approaches embedded in planning cultures should be tested within specific urban contexts.

## 4 Conclusion

This paper tracks the rise of urban climate change experimentation as a new means through which climate governance is conducted. The survey shows that experimentation has been a growing trend after the Kyoto ratification in 2005 and it is not confined to specific regions. Its emergence cannot simply be predicted by the general characteristics of the city (whether this is size, density or wealth) or the city's commitments to climate change action. Among all the factors considered, the internationalisation of urban environmental governance through city networks will need closer attention in further research.

Experimentation involves multiple forms of technical and social innovation. Despite the diversity of experiments, these do not always challenge established ideas about the management of resources in the city. For example, in the case of interventions on energy system there is still a separation between interventions seeking to reconfigure consumption patterns, mostly in the built environment, and interventions seeking to transform the systems of energy production. Experiments in energy decentralisation and in energy production within the household question this divide, but the survey data suggest that such radical experiments – capable to foster systemic change – coexist with forms of experimentation that do not fundamentally challenge mainstream ideas about the production and consumption of energy in the city. Further research is needed to examine the potential to move from incremental interventions (like the majority included in this survey) to interventions leading towards systemic change.

While local governments lead the majority of experiments, many other actors intervene either leading experiments or in partnerships. Partnership emerges as a key feature in climate change governance. Linked to enabling modes of governance it emphasises the extension of local forms of authority through the support of initiatives conducted by non-state actors. Another interesting feature is the inclusion of justice claims in climate change experiments, especially among private and civil society actors (rather than local governments), who may need to construct explicitly justifications for their attempts to govern climate change.

Finally, the analysis throws interesting questions regarding the emergence of a characteristic form of urban climate change experimentation in Asia. In particular, the analysis suggests that experiments where private actors intervene in urban infrastructure predominate in Asia, in contrast to other regions where neither a particular sector nor particular actors appear to predominate. This new trend of purposive experimentation in climate change governance in cities in Asia, could be associated with new private-led forms of urbanism in emerging economies or with different cultural approaches to managing climate change.

This methodology has allowed, for the first time, a systematic comparison of urban climate change experiments across 100 cities. The long-term effectiveness of experiments and their interaction across scales are issues beyond the scope of this analysis to be addressed with further research. However, alongside case-study based research, this methodology provides a fruitful avenue to understand urban climate change experimentation in context. Revealing the underlying drivers in climate change experimentation, factors hindering action, effectiveness on the ground and impact could be further developed through additional survey work, focused on specific regions or metropolitan areas. Overall, the methodology reveals the heterogeneity and ubiquity of climate change experimentation and traces the opening up of new spaces for climate change governance in the city.

## Figures and Tables

**Table 1:** List of 100 sample cities.

Urban area	Country
Addis Ababa	Ethiopia
Ankara	Turkey
Athens	Greece
Atlanta	USA
Baghdad	Iraq
Bangalore	India
Bangkok	Thailand
Barcelona	Spain
Beijing	China
Belo Horizonte	Brazil
Berlin	Germany
Birmingham	UK
Bogota	Colombia
Boston	USA
Budapest	Hungary
Buenos Aires	Argentina
Cairo	Egypt
Cape Town	South Africa
Caracas	Venezuela
Chennai	India
Chicago	USA
Dallas/Fort Worth	USA
Delhi	India
Denver	USA
Detroit	USA
Dhaka	Bangladesh
Fukuoka	Japan
Guadalajara	Mexico
Hamburg	Germany
Hanoi	Vietnam
Ho Chi Minh City	Vietnam
Hong Kong	China
Houston	USA
Hyderabad	India
Istanbul	Turkey
Jakarta	Indonesia
Jeddah	Saudi Arabia
Johannesburg/East Rand	South Africa
Karachi	Pakistan
Khartoum	Sudan
Kinshasa	Congo
Kolkata	India
Kuala Lumpur	Malaysia
Lagos	Nigeria
Lahore	Pakistan
Lima	Peru
Lisbon	Portugal
London	UK
Los Angeles	USA
Madrid	Spain
Manchester	UK
Manila	Philippines
Melbourne	Australia
Mexico City	Mexico
Miami	USA
Milan	Italy
Minneapolis/St. Paul	USA
Monterey	Mexico
Montreal	Canada
Moscow	Russia
Mumbai	India
Munich	Germany
Nagoya	Japan
Naples	Italy
New York	USA
Osaka/Kobe/Kyoto	Japan
Paris	France
Philadelphia	USA
Phoenix/Mesa	USA
Porto Alegre	Brazil
Quito	Ecuador
Recife	Brazil
Rio de Janeiro	Brazil
Riyadh	Saudi Arabia
Rome	Italy
Rotterdam	Netherlands
San Diego	USA
San Francisco/Oakland	USA
Santiago	Chile
Sao Paulo	Brazil
Seattle	USA
Seoul/Incheon	South Korea
Shanghai	China
Shenyang	China
Shenzhen	China
Singapore	Singapore
St. Petersburg	Russia
Stockholm	Sweden
Sydney	Australia
Taipei	Taiwan
Tampa/St. Petersburg	USA
Tehran	Iran
Tel Aviv	Israel
Tianjin	China
Tokyo/Yokohama	Japan
Toronto	Canada
Vancouver	Canada
Vienna	Austria
Warsaw	Poland
Washington, DC	USA

**Table 2:** Key descriptors for the city sample. Data from the World Majors Website (http://www.citymayors.com/, accessed 07.07.12).

	Minimum value	Maximum value	Mean	Std. deviation
Population in 2006 (million)	1.3	33.2	6.1	5.0
Land area in 2006 (km^2^)	304	8683	1507.52	1463.7
Density in 2006(people/km^2^)	700	29,650	6330.87	5497.2
Gross domestic product (US$Bn) in 2005	7	1191	150.6	183.2
Gross domestic product per capita (UC$/person)	1818.2	76,004.07	28,127.1	20,732.3
World City Rank^a^	1	11	6.7	3.1
Annual Population Growth	−.68	4.44	1.26	1.10

a See Beaverstock et al. (1999).

**Table 3:** Types of schemes included in each sector (adapted from UN-Habitat, 2011).

Objective in relation to climate change	Sector	Types of schemes
Mitigation	Urban infrastructure	Alternative energy supply (renewable or low carbon)
		Landfill gas capture
		Alternative water supply
		Collection of waste for recycling and reuse
		Energy and water conservation measures
		Network demand reduction measures
	Built environment	Use of energy-efficient materials
		Energy-efficient design
		Building-integrated alternative energy supply
		Building-integrated alternative water supply
		New-built energy and water-efficient technologies
		Retrofitting energy and water-efficient technologies
		Energy and water-efficient appliances
		Building-integrated demand reduction measures
	Urban form	Urban expansion and suburban development
		New urban development
		Reuse of Brownfield land
		Neighbourhood and small-scale urban renewal
	Transport	New low-carbon transport infrastructure
		Low-carbon infrastructure renewal
		Fleet replacement
		Fuel switching
		Enhancing energy efficiency
		Mobility demand reduction measures (reducing travel)
		Mobility demand enhancement measures (alternative means of travel)
	Carbon sequestration	Urban capture and storage
		Urban tree-planting programmes
		Restoration of carbon sinks
		Preservation and conservation of carbon sinks
		Carbon offset schemes

Adaptation		Cooling services and designs
		Measures securing energy and water supply
		Flood protection
		Bushfire protection
		Relocation and zoning policies
		Blue and green infrastructure
		Building codes for extreme weather
		Early warning systems
		Behaviour-based measures

**Table 4:** Categories for database design.

Overall question	Indicators	Definition
Where and when a climate change experiment occurs	Location	Name of urban area and geographical regions
	Dates	Starting date and date of reported completion if stated
	Urban character	Statement of the urban character of the experiment

What are these experiments how are they developed	Type of experiment	Classification in sectors
	Objectives	Statement of objectives, completion indicators and milestones
	Type of innovation	Reported forms of innovation including new technologies, designs, social and policy innovations
	Institutional factors	Factors which contributed to the success of the experiment or hinder its development as reported
	Sector specific information	Record of interventions in different systems of provision; specification of technologies involved; record of services met in each experiment

Who leads initiatives and how they are governed	Actors involved	Initiating actors, partners, donors, supporters
	Funding	Total funding available and source of funding
	Mode of governance	How the initiative is achieved (self-governing, regulation, enabling, provision)
	Environmental justice	Is environmental justice considered?

**Table 5:** Contingency table for the distribution of initiatives in different sectors, in more and less developed regions and in different periods (expected frequencies in brackets).

	When			Where		
	Pre-Kyoto agreement	Pre-Kyoto ratification	Post-Kyoto	Less developed countries	More developed countries	Total
Adaptation	4 (4)	7 (12)	65 (60)	36 (36)	40 (40)	76
Built environment	8 (8)	33 (24)	114 (122)	59 (74)	96 (81)	155
Carbon sequestration	2 (2)	3 (5)	30 (28)	24 (16)	11 (18)	35
Transport	6 (6)	18 (19)	94 (93)	59 (56)	59 (62)	118
Urban form	5 (2)	8 (7)	29 (33)	16 (20)	26 (22)	42
Urban infrastructure	8 (10)	30 (32)	163 (159)	105 (96)	96 (105)	201

Total	33	99	495	299	328	627

**Table 6:** Contingency table for the form of innovation in different sectors (expected frequencies in brackets).

	Innovation is…			Total
	Social	Technical	Both	
Adaptation	30 (18)	41 (38)	5 (20)	76
Built environment	37 (37)	67 (77)	51 (41)	155
Carbon sequestration	19 (8)	10 (17)	6 (9)	35
Transport	31 (28)	57 (59)	30 (31)	118
Urban form	8 (10)	14 (21)	20 (11)	42
Urban infrastructure	24 (48)	123 (100)	54 (53)	201

Total	149	312	166	627

**Table 7:** Summary data table of climate change experiments in different urban sectors.

Sector	Focus on energy	Consumption	Production	Total
Built environment	116 (75%)	101	15	155
Urban form	9 (21%)	5	4	42
Urban infrastructure	156 (78%)	55	101	201

Total	281	161	120	627

**Table 8:** Cross tabulation for when, where and what experiments are led by (local government, other public organism, private actors or civil society organisations).

	Leading actor	Local government	Other government	Private	Civil society	Grand total
Where	Africa	29	6	4	2	41
	Asia	86	13	51	12	162
	Europe	112	11	24	12	159
	North America	102	11	9	14	136
	Oceania	15	1	0	1	17
	South and Central America	69	24	9	10	112

When	Pre-Kyoto agreement	20	4	4	5	33
	Pre-Kyoto ratification	65	8	15	11	99
	Post-Kyoto	328	54	78	35	495

What	Adaptation	46	19	4	7	76
	Built environment	101	13	23	18	155
	Carbon sequestration	16	8	5	6	35
	Transport	96	10	7	5	118
	Urban form	27	4	5	6	42
	Urban infrastructure-waste	18	2	13	1	34
	Urban infrastructure-water	10	0	1	0	11
	Urban infrastructure-energy	99	10	39	8	156

Total	Grand total	413	66	97	51	627

**Table 9:** Contingency table for the distribution of initiatives in relation to different forms of partnership (expected frequencies in brackets).

Leading	Partner					Total
	Civil society	Local government	Private	Other government	No partnership	
Civil society	5 (5.1)	18 (3.4)	8 (13.7)	2 (1.8)	18 (26.9)	51
Local government	44 (41.5)	4 (27.7)	112 (111.3)	14 (14.5)	239 (218.0)	413
Other government	8 (6.6)	12 (4.4)	12 (17.8)	0 (2.3)	34 (34.8)	66
Private	6 (9.7)	8 (6.5)	37 (26.1)	6 (3.4)	40 (51.2)	97

Total	63	42	169	22	331	627

**Table 10:** Contingency table for the distribution of initiatives in terms of leading actor, partnerships and mode of governance (expected frequencies in brackets).

	Mode of governance				Total
	Enabling	Provision	Regulation (hard and soft)	Self-governing	
*Leading actor*					
Civil society	26 (16.8)	15 (20.9)	3 (7.7)	7 (8.0)	51
Local government	117 (125.8)	160 (169.3)	74 (58.0)	62 (59.9)	413
Other government	22 (18.9)	29 (27.0)	9 (8.7)	6 (9.0)	66
Private	26 (29.5)	53 (39.8)	2 (13.6)	16 (14.1)	97

*Partnership*					
No	87 (100)	134 (135)	66 (46)	43 (48)	330
Yes	104 (90)	123 (121)	22 (41)	48 (43)	297

*Total*					
	191	257	88	91	627

**Table 11:** Contingency table for the consideration of environmental justice in different sectors (expected frequencies in brackets).

	Justice considered		Total
	N	Y	
*Actor*			
Private	67 (73)	30 (23)	97
Public	380 (361)	99 (117)	479
Civil society	26 (38)	25 (12)	51

Total	473	154	627
